# Noise exposure burden and hearing protection compliance among Saudi military personnel: A cross-sectional study

**DOI:** 10.4102/sajcd.v73i1.1170

**Published:** 2026-04-09

**Authors:** Ahmad A. Alanazi, AlHanouf N. Alhathal, Abrar M. Almutairi, Nada S. Alrawdhan, Maryam A. Alrashied

**Affiliations:** 1 Department of Audiology and Speech Pathology, College of Applied Medical Sciences, King Saud bin Abdulaziz University for Health Sciences, Riyadh, Saudi Arabia; 2 King Abdullah International Medical Research Center, Riyadh, Saudi Arabia; 3 Ministry of National Guard Health Affairs, Riyadh, Saudi Arabia; 4 Research Unit, College of Applied Medical Sciences, King Saud bin Abdulaziz University for Health Sciences, Riyadh, Saudi Arabia

**Keywords:** awareness, hearing loss, hearing protection, hearing conservation, military, noise-induced hearing loss, safety, Saudi Arabia

## Abstract

**Background:**

Noise-induced hearing loss (NIHL) results from prolonged or intense exposure to loud sounds, causing permanent auditory damage often accompanied by tinnitus, reduced hearing sensitivity and difficulty understanding speech. Use of appropriate hearing protection is essential to minimizing exposure, preventing cochlear injury and reducing the long-term impact of NIHL.

**Objectives:**

This study aimed to assess noise exposure experiences, their effects on hearing sensitivity and the use of hearing protection among Saudi military personnel.

**Methods:**

This cross-sectional study used a self-administered Arabic questionnaire developed after a literature review and consultation with a focus group. Copies were distributed to military personnel visiting the audiology clinic at King Abdulaziz Medical City in Riyadh from 2024 to 2025 and shared via social media nationwide. The questionnaire included 14 items across three sections: demographics, experience with noise exposure and hearing protection and awareness of noise-related impacts.

**Results:**

A total of 256 participants completed the questionnaire; the majority were male (97.3%), lived in Riyadh (85.2%), and were aged 31 years–40 years with a high school education. Most reported no hearing loss across years of experience and exposure to loud sound sources. Notably, 130 participants (50.8%) did not use hearing protection despite being aware that loud noise can cause hearing loss and tinnitus (*p* = 0.01). Significant associations were found between reported hearing loss and involvement in shooting or use of weapons (*p* = 0.01) as well as noise source types (*p* = 0.02).

**Conclusion:**

Although most participants were aware of the harmful effects of loud noise and related symptoms, only one-third consistently used hearing protection. Training to enhance awareness of NIHL risks, along with periodic hearing assessments for early detection, is recommended.

**Contribution:**

This study highlights a gap between awareness of noise-induced hearing loss and the use of hearing protection among Saudi military personnel. It identifies weapon-related noise exposure as associated with reported hearing loss, emphasizing the need for targeted hearing conservation strategies.

## Introduction

Noise-induced hearing loss (NIHL) results from exposure to excessively loud sounds and may cause a short-term reduction in hearing sensitivity that recovers after hours or days of rest (i.e. temporary threshold shift) or irreversible hearing loss (i.e. permanent threshold shift) (Ryan et al., 2016). The impact on the auditory system may present in various ways, depending on the type, duration and intensity of the noise exposure. Damage to hair cells in the cochlea and other inner structures can be caused by acoustic exposure, including intense impulse and/or multiple recurring exposures or listening for an extended period (Wada et al., 2017). Although NIHL is predictable and preventable by wearing hearing protection, irreversible damage to the cochlear hair cells can occur (Alanazi et al., 2025). According to the World Health Organization (WHO), nearly 10% of people worldwide are exposed to excessive noise, and around 5.3% of the global population is affected by NIHL (Themann & Masterson, 2019).

Occupational NIHL, the most prevalent occupational disease in the world, affects workers in an environment that is most likely to encounter an impulse noise or a long period of loud sounds (Chen et al., 2020). Noise-induced hearing loss and tinnitus are commonly reported symptoms among individuals who are exposed to occupational, environmental and recreational noise, including military personnel (Alanazi et al., 2025; Yankaskas, 2013). Some studies have reported that during their careers, almost every personnel, regardless of their military branch, is exposed to high intensity sounds (Collee et al., 2011; Yankaskas, 2013). Occupational NIHL can be prevented by wearing hearing protection, as it decreases the incidence of acoustic injury (Alanazi et al., 2025). Unfortunately, a significant number of employees who are at risk of acoustic injury are not willing to use hearing protection because of the lack of knowledge of NIHL and its effects (Abel, 2008).

The literature regarding this topic is still limited in Saudi Arabia. A few studies investigated NIHL and awareness of noise exposure among the general population. A survey study by Alzahrani et al. (2023) conducted in Makkah, Saudi Arabia, assessed awareness of NIHL and its association with personal listening devices (PLDs). The study revealed that 22% of 384 participants, who were mostly males, had mild-to-severe hearing impairment, and approximately 40% of them were involved in work-related noise. Duration and level of listening to PLDs were additional risk factors. The study suggested a need for more NIHL awareness initiatives. A study by Alenzi et al. (2024) conducted in Al-Jouf, Northern Saudi Arabia, examined public awareness of NIHL related to the use of headphones. Although the study showed that 54.1% of 438 participants acknowledged that noise-induced hearing problems were preventable, a lack of awareness and knowledge about NIHL and its symptoms was apparent. Another study, conducted by Alzahrani et al. (2024), included 400 participants from southern Saudi Arabia and evaluated the symptoms, knowledge, beliefs and preventive practices regarding NIHL. Their results showed that tinnitus and unclear speech were common symptoms, with a frequent need to increase the volume of the radio and television. Most of their participants knew that high noise affects hearing, but only 9.5% knew the harmful exposure duration. AlYahya et al. (2023) evaluated auditory symptoms induced by exposure to noise, acceptance of wearing ear protection equipment and the duration of noise exposure among the general population of the Eastern Region of Saudi Arabia. Their study showed a low level of knowledge and awareness of NIHL and the importance of hearing protection, with the need for more health campaigns to raise awareness of NIHL. AlQahtani et al. (2022) examined the relationship between NIHL and the use of headphones in Hail, Saudi Arabia. They found a low level of awareness regarding hearing loss and its causes, with more than half of the participants engaging in unhealthy listening habits using their devices.

Noise-induced hearing loss studies among different military populations have been well established. However, differences in exposure type, assessment methods, population characteristics, study design, noise quantification and hearing protection practices can all influence reported rates and severity of NIHL in military studies, making direct comparisons challenging. For example, Coluk et al. (2025) found significantly higher hearing thresholds in Turkish special police forces compared to a healthy control group. Lachaux et al. (2024) highlighted that the prevalence of long-term hearing impairment after acute acoustic trauma in the French military exceeded 20%. Luha et al. (2020) found that half of their participants from Estonian defence forces personnel reported hearing loss during their service period associated with non-use of hearing protection.

In Saudi Arabia, the literature of NIHL among military personnel is scant, with only a few published studies. Alanazi et al. (2025) investigated the effects of noise exposure on military personnel in Riyadh, Saudi Arabia. They found that bilateral sensorineural hearing loss and tinnitus were more prevalent and reported that limited awareness of NIHL, inadequate training on the use of hearing protection devices and the absence of stringent enforcement of protective protocols were the factors leading to NIHL. Another study by Alsaab et al. (2021) examined the prevalence of NIHL, awareness, hearing impairment and protection of hearing among military personnel in the Eastern Region of Saudi Arabia. This study collected data via a questionnaire and pure tone audiometry. It revealed that more than half of the participants were unaware of the consequences of noise exposure, and none used proper hearing protection. Furthermore, more than half of the participants had symptoms because of NIHL, such as tinnitus. From the previously published studies, the investigation of NIHL among military personnel was limited to two regions (i.e., Riyadh and the Eastern Region) of Saudi Arabia. No study investigated the awareness of NIHL among military personnel across all regions in Saudi Arabia. Therefore, our study aimed to measure the history of noise exposure, its effects on hearing sensitivity and the use of hearing protection among military personnel in all regions of the country.

## Research methods and design

This cross-sectional descriptive study was designed to determine the awareness of NIHL among military personnel in Saudi Arabia. This study was conducted in Riyadh, Saudi Arabia. The inclusion criteria comprised all Saudi military personnel who were exposed to weapon noises and who were aged 18 years or older at the time of completing the questionnaire.

### The questionnaire

Arabic is the primary language in Saudi Arabia, so a self-administered Arabic questionnaire was developed for data collection after a review of the literature and consultation with a focus group, including all the authors. This questionnaire consisted of 14 questions distributed into three sections: (1) demographic information (six questions), (2) experience with noise exposure and hearing protection (five questions) and (3) awareness of the impacts of noise exposure (three questions) (Appendix 1). The expert group identified and resolved any inadequate expressions or concepts that may affect the understanding of the questions. This questionnaire was piloted on 10 participants and presented as hard copies. The internal consistency of the questionnaire was evaluated using Cronbach’s alpha (α), while construct validity was examined through principal component analysis (PCA) to explore the underlying factor structure of the instrument. A Cronbach’s α score of 0.73 was obtained while PCA supported a multi-component structure with satisfactory variance explained and appropriate item loadings. The data were analysed using Statistical Package for Social Sciences (SPSS) for Windows version 29.0 (IBM SPSS Statistics, IBM Corporation, Armonk, NY, United States [US]). Statistical analyses were conducted using Chi-square tests for all categorical variables. Missing data were handled via listwise deletion. A significance level of *p* < 0.05 was applied, and all reported *p*-values corresponded to the Chi-square analyses. Informed consent was obtained from all participants before filling out the pilot questionnaire. The pilot study participants were asked about the clarity of all questions to ensure that no question was misunderstood. They had an excellent understanding of the questions, and no comments were received. Accordingly, the content of the questionnaire was not modified. These participants were not included in the sample size of the main study because they were involved in the pilot phase (before the final sample size calculation), which was conducted to assess feasibility, clarity of procedures and questionnaire comprehension. Including pilot participants in the main analysis could introduce bias because of prior exposure to the study instruments.

A convenience sampling technique was used in this study. The sample size was calculated to be 384 participants using Raosoft sample size calculator (Raosoft, Inc., US) with 5% margin of error, 95% confidence level and 50% response distribution. The questionnaire was self-administered and distributed to all military personnel who visited the audiology clinic at King Abdulaziz Medical City in Riyadh, Saudi Arabia. Additionally, the questionnaire was prepared electronically on Google Forms (Google LLC, Mountain View, California, US) and distributed to reach many military personnel all over Saudi Arabia in a time-efficient and cost-effective way. The link to the questionnaire was posted on and shared via social media (X, Facebook, Telegram and WhatsApp). Electronic informed consent was obtained in the study as well. The questionnaire link was accessible for 1 year from 2024 to 2025, which is the period during which participants were recruited. The link was reposted multiple times on social media. Participation was voluntary, and answers were anonymous. Although distributing the questionnaire via social media may reduce control over the sampling process and result in responses from individuals outside the intended target population, as well as introduce self-selection bias, clear inclusion and exclusion criteria were stated at the beginning of the questionnaire and screening questions were used to identify eligible participants. Responses that did not meet the predefined eligibility criteria were excluded from the analysis. The dual distribution approach (i.e. hard and soft copies of the questionnaire) was intentionally used to maximise reach and inclusivity. Hardcopy questionnaires allowed access to participants with limited digital access or lower familiarity with online platforms, while the digital version facilitated rapid dissemination and convenience for participants comfortable with online participation. Using both modes helped improve response rates, ensured broader coverage of the target population and reduced potential sampling bias associated with relying on a single distribution method. To minimise duplicate responses in the online survey, each participant could complete the survey only once per unique email or device and data were checked for duplicate entries during data cleaning.

### Ethical considerations

Ethical clearance to conduct this study was obtained from the King Abdullah International Medical Research Center, Institutional Review Board (No. #NRC22R/488/10). No identifiable or health information was collected. Only the authors had access to the data from the completed questionnaires.

## Results

A total of 256 participants were included in this study. The majority of participants (41.4%) were aged 31 years–40 years. Most of the participants (97.3%) were male. The education level showed that 38.3% of them had completed high school, while those who had a diploma or a bachelor’s degree were 20.3% and 22.3%, respectively. Most participants had no medical educational background, representing 78.1%. Riyadh was the region where the majority of the participants lived, accounting for 85.2%. The majority of participants (41%) had 16 years or more of experience (Table 1).

**TABLE 1:** Demographic characteristics of the participants.

Variable	Frequency	
	*n*	%
**Age (years)**		
18–30	72	28.1
31–40	106	41.4
41–50	56	21.9
51–60	22	8.6
**Gender**		
Male	249	97.3
Female	7	2.7
**Education**		
Intermediate school	8	3.1
High school	98	38.3
Diploma	52	20.3
Bachelor	57	22.3
Master	33	12.9
Others (e.g. Saudi Medical Board)	8	3.1
**Personnel with a medical education background**		
Yes	56	21.9
No	200	78.1
**Region**		
Riyadh	218	85.2
Makkah	3	1.2
Eastern	1	0.4
Qassim	3	1.2
Northern borders	29	11.3
Jazan	2	0.8
**Experience (years)**		
1–4	32	12.5
5–9	54	21.1
10–15	65	25.4
≥ 16	105	41.0

Table 2 shows the survey questions about the experiences of hearing and noise exposure among the participants. Most of the participants (76.6%) have not had their hearing tested. Additionally, 206 (80.5%) participants reported not having hearing loss. Most participants (*n* = 185, 72.3%) were participating in using weapons and shootings. Exposure to loud noises during military service ranged among the participants. Of the total participants, 25.8% (*n* = 66) were exposed to loud noise sources for 5 years or more, while 17.2% (*n* = 44) were exposed to loud noise sources for less than a year. Most participants (*n* = 132, 51.6%) were exposed to light weapons. Surprisingly, 130 (50.8%) participants did not use any hearing protection while being exposed to loud noise sources. In contrast, 182 (71.1%) participants and 202 (78.9%) participants were aware that exposure to loud noises can cause hearing loss and tinnitus, respectively.

**TABLE 2:** Hearing and noise exposure experiences of the participants.

Experience of hearing and noise exposure	Frequency	
	*n*	%
**Have you ever had your hearing tested?**		
Yes	60	23.4
No	196	76.6
**Do you have hearing loss?**		
Yes	50	19.5
No	206	80.5
**Are you currently participating in shooting and/or using weapons?**		
Yes	185	72.3
No	71	27.7
**How long have you been exposed to loud noises during military service (years)?**		
< 1	44	17.2
1–2	38	14.8
3–4	29	11.3
≥ 5	66	25.8
**What type of loud noise sources did you expose yourself to?**		
Heavy weapons	55	21.5
Light weapons	132	51.6
Helicopters	15	5.9
Not applicable	54	21.1
**Do or did you use hearing protection while shooting or when exposed to loud noises?**		
Yes	83	32.4
No	130	50.8
Not applicable	43	16.8
**Do you know that exposure to loud noises can cause hearing loss?**		
Yes	182	71.1
No	74	28.9
**Do you know that exposure to loud noises can cause tinnitus?**		
Yes	202	78.9
No	54	21.1

The association between the level of awareness of exposure to loud noises and its potential to cause hearing loss and tinnitus is shown in Table 3. There is no significant correlation between age and the awareness that exposure to loud noises can cause hearing loss (*p* = 0.41) and tinnitus (*p* = 0.40). Of the total participants, the age group of 31 years–40 years had a higher awareness of the effects of exposure to loud noises on hearing and balance. There is a statistically significant association between participants’ education and awareness that loud noises can cause tinnitus (*p* = 0.04). However, no statistically significant association was observed among participants with education and awareness that loud noises can cause hearing loss (*p* = 0.47). Moreover, no statistically significant associations were observed between those who had their hearing tested and their awareness that loud noises can cause hearing loss (*p* = 0.15) and tinnitus (*p* = 0.18). No statistically significant associations were found between having a medical background and awareness that loud noises can cause hearing loss (*p* = 0.32) and tinnitus (*p* = 0.94). The association between the use of hearing protection during exposure to loud noises and the awareness that loud noises can cause hearing loss and tinnitus was statistically significant (*p* = 0.01).

**TABLE 3:** Association between the awareness of noise exposure effects and other variables.

Variables	Awareness level regarding the effects of loud noise exposure on the auditory system									
	Tinnitus					Hearing loss				
	Yes		No		*p*-value	Yes		No		*p*-value
	*n*	%	*n*	%		*n*	%	*n*	%	
**Age (years)**										
18–30	53	26.2	19	35.2	0.40	47	25.8	25	33.8	0.41
31–40	84	41.6	22	40.7	-	75	41.2	31	41.9	-
41–50	48	23.8	8	14.8	-	42	23.1	14	18.9	-
51–60	17	8.4	5	9.3	-	18	9.9	4	5.4	-
**Education**										
Intermediate school	8	4.0	0	0.0	0.04	7	3.8	1	1.4	0.47
High school	69	34.2	29	53.7	-	64	35.2	34	45.9	-
Diploma	41	20.3	11	20.4	-	38	20.9	14	18.9	-
Bachelor	46	22.8	11	20.4	-	45	24.7	12	18.9	-
Master	31	15.3	2	3.7	-	22	12.1	11	14.9	-
Others (e.g. Saudi Medical Board)	7	3.5	1	1.9	-	6	3.3	2	2.7	-
**Have you ever had your hearing tested?**										
Yes	51	25.2	9	16.7	0.18	47	25.8	13	17.6	0.15
No	151	74.8	45	83.3	-	135	74.2	61	82.4	-
**Personnel with a medical background**										
Yes	43	21.8	12	22.2	0.94	43	23.6	13	17.6	0.32
No	158	78.2	42	77.8	-	139	76.4	61	82.4	-
**Do you use hearing protection while shooting or when exposed to loud noises?**										
Yes	77	45.8	6	13.3	0.01*	74	49.0	9	14.5	0.01*
No	91	54.2	39	86.7	-	77	51.0	53	85.5	-

* , Statistically significant.

Table 4 illustrates the association between hearing loss and other related variables. Of the total participants, 88% were participating in shooting or using weapons. This was statistically correlated with the presence of hearing loss (*p* = 0.01). The association between the participants’ experience of being exposed to loud noises during military service and the presence of hearing loss was also statistically significant (*p* = 0.04). There was a statistically significant association between the type of loud noise sources the participants were exposed to and the presence of hearing loss (*p* = 0.02).

**TABLE 4:** Association between hearing loss and other variables.

Variables	Do you have hearing loss?				*p*-value
	Yes		No		
	*n*	%	*n*	%	
**Are you currently participating in shooting and/or using weapons?**					
Yes	44	88.0	141	68.4	0.01*
No	6	12.0	65	31.6	-
**How long have you been exposed to loud noises during military service (years)?**					
≤ 4	111	62.7	90	69.2	0.04*
> 4	66	37.3	48	34.8	-
**What type of loud noise sources did you expose yourself to?**					
Heavy weapons	18	36.0	37	18.0	0.02*
Light weapons	24	48.0	108	52.4	-
Helicopters	3	6.0	12	5.8	-

* , Statistically significant.

## Discussion

The experience of noise exposure among military personnel in Saudi Arabia reflects global trends observed in military populations regarding NIHL. The nature of their service often involves exposure to high-decibel environments, such as firearms training and explosions, which significantly increases their risk of auditory damage. Our study measured the experience of noise exposure, its effects on hearing sensitivity and the use of hearing protection among military personnel. The findings provide valuable insights into the demographic characteristics of the participants, their exposure to noise and their awareness of noise-induced hearing health risks. The majority of participants (41.4%) were aged 31 years–40 years, with a significant male predominance (97.3%). This demographic distribution aligns with previous studies on military personnel, where males and individuals in their 30s commonly represent the majority of military personnel (Alsaab et al., 2021). Lie et al. (2016), in their systematic review on occupational NIHL, reported that men are more likely to experience greater hearing loss compared to women. Educational attainment varied, with a notable proportion (38.3%) having only a high school education. A large majority (78.1%) lacked a medical background, which may have implications for their understanding of health risks associated with noise exposure.

Over half (51.6%) were exposed to light weapons, and nearly a quarter (25.8%) reported prolonged exposure to loud noises for 5 years or more. Alarmingly, more than half (50.8%) of the participants did not use hearing protection during such exposures, which puts them at high risk of hearing loss and tinnitus. The two most common service-connected disabilities among US veterans were tinnitus and hearing loss (U.S. Department of Veterans Affairs, 2024). The behaviour of not using hearing protection aligns with findings in similar populations, where the use of hearing protection is often under-prioritised despite widespread knowledge of its importance. Studies have highlighted a lack of consistent hearing protection usage and limited awareness of the long-term consequences of noise exposure among military personnel (Basheer et al., 2019; Themann & Masterson, 2019). A study among the military personnel in the Eastern Region of Saudi Arabia indicated that more than half of them were unaware of the impacts of noise exposure, with no appropriate use of hearing protection (Alsaab et al., 2021). Another study on the general population in the Eastern Region of Saudi Arabia revealed that the young age group showed a low level of knowledge and a low level of awareness of NIHL and the importance of earplugs (AlYahya et al., 2023). Awareness of the potential consequences of loud noise exposure was relatively high, with 71.1% and 78.9% of participants acknowledging its potential to cause hearing loss and tinnitus, respectively. However, the gap between awareness and protective behaviour underscores a critical area for intervention. A study among the general population in Southern Saudi Arabia depicted gaps in knowledge about the intensity and duration of sound that negatively affect hearing (Alzahrani et al., 2024).

The statistical analyses revealed several significant associations. Participants with higher education levels demonstrated greater awareness of the risk of tinnitus caused by noise exposure (*p* = 0.04). This finding underscores the potential role of education in enhancing awareness, a factor echoed in existing research advocating targeted educational initiatives to promote hearing health (Alnuman & Ghnimat, 2019). Additionally, those who used hearing protection were significantly more likely to be aware of the risks of noise exposure (*p* = 0.01). These results suggest a reciprocal relationship, where awareness fosters protective behaviour, which in turn, reinforces awareness. Exposure to loud noises during military service and current involvement in shooting activities were significantly associated with the presence of hearing loss (*p* = 0.01). This finding supports extensive evidence linking military noise exposure with auditory dysfunction, including permanent threshold shifts and tinnitus (Yong & Wang, 2015). The type of noise source also emerged as a significant factor (*p* = 0.02), further emphasising the need to tailor interventions based on specific exposures.

Surprisingly, no statistically significant associations were found between participants’ medical background and their awareness of noise-induced hearing risks. This may indicate that general health knowledge does not necessarily extend to specialised awareness of auditory health risks, reinforcing the importance of targeted training programmes. Similarly, no significant associations were found between age or prior hearing tests and awareness levels, suggesting that these factors alone are insufficient to drive awareness. Alanazi et al. (2025) urged the implementation of robust hearing conservation programmes, encompassing routine auditory evaluations, enforced use of hearing protection and comprehensive education on the risks of NIHL among Saudi military personnel. Verbeek et al. (2014) suggested that strengthening the enforcement of legislation and improving the implementation of hearing loss prevention programmes can help lower workplace noise levels, in addition to conducting thorough assessments of technical interventions and their long-term impacts.

### Limitations and future research

Although participation in the study was allowed for approximately a year, only 256 individuals participated in the study, which is below the minimum effective sample size. Most of the participants were from one region, Riyadh. The questionnaire did not include questions about the type of hearing protection the participants used. No control for other confounders, such as age-related hearing loss and diseases (e.g. diabetes mellitus), was applied. The questionnaire was distributed via social media platforms, which limited control over the sampling process. This distribution method may have allowed individuals outside the intended target population to access the survey and introduced self-selection bias, potentially affecting the representativeness and generalisability of the findings. Although eligibility criteria and screening questions were used to exclude ineligible responses, residual sampling bias cannot be fully ruled out. Future research should explore the specific experiences and compliance barriers within the Saudi military to design culturally and contextually relevant hearing conservation strategies. Future research should also evaluate the effectiveness of intervention strategies in reducing NIHL in this high-risk population.

## Conclusion

This study highlights critical gaps in hearing protection behaviours among military personnel despite relatively high awareness levels of the risks associated with loud noise exposure. Educational attainment and the use of hearing protection were significant factors influencing awareness, while prolonged noise exposure and participation in shooting activities were strongly associated with hearing loss. These findings underline the need for comprehensive auditory health programmes in military settings, focusing on improving the adoption of protective measures and increasing awareness among personnel with varying educational backgrounds. Incorporating structured training programmes to improve awareness of the risks of NIHL and the importance of hearing protection is recommended. Periodic hearing assessments to identify early signs of hearing damage and ensure timely intervention are also recommended. Finally, hearing protection devices specifically designed for military contexts should be available and used effectively.

## Appendix 1

### Questionnaire

#### A. Demographic information

1. What is your age (years)?
  - 18–30
  - 31–40
  - 41–50
  - > 50
2. What is your gender?
  - Male
  - Female
3. What is your highest level of education?
  - Intermediate school
  - High school
  - Diploma
  - Bachelor
  - Master
  - Doctorate
  - Other (please specify)
4. What is your service duration during the military services (years)?
  - 1–4
  - 5–9
  - 10–15
  - ≥ 16
5. In which region do you work?
  - Riyadh
  - Eastern Region
  - Tabuk
  - Northern Borders
  - Jawf
  - Makkah
  - Madinah
  - Bahah
  - Jizan
  - Ha’il
  - Asir
  - Qasim
  - Najran
6. Are you a medical professional?
  - Yes
  - No

#### B. Experience with noise exposure and hearing protection

7. Did you have your hearing checked?
  - Yes
  - No
8. Do you have hearing loss?
  - Yes
  - No
9. Do you participate or have you participated in shooting activities?
  - Yes
  - No (Choose not applicable for questions 10–12)
10. How long did you expose yourself to loud noises (years)?
  - < 1
  - 1–2
  - 3–4
  - ≥ 5
  - Not applicable
11. What type of weapon do you use or have you used?
  - Light weapons (e.g. rifles and pistols)
  - Heavy weapons (e.g. tanks and rockets)
  - Attack helicopters
  - Other (specify)
  - Not applicable

#### C. Awareness of the impacts of noise exposure

12. Do you use or have you used hearing protection during shooting activities?
  - Yes
  - No
13. Do you know that exposure to loud noises can cause hearing loss?
  - Yes
  - No
14. Do you know that exposure to loud noises can cause tinnitus?
  - Yes
  - No
